# The positive prognostic effect of stromal CD8+ tumor-infiltrating T cells is restrained by the expression of HLA-E in non-small cell lung carcinoma

**DOI:** 10.18632/oncotarget.6506

**Published:** 2015-12-02

**Authors:** Mehrdad Talebian Yazdi, Sander van Riet, Annemarie van Schadewijk, Marta Fiocco, Thorbald van Hall, Christian Taube, Pieter S. Hiemstra, Sjoerd H. van der burg

**Affiliations:** ^1^ Department of Pulmonology, Leiden University Medical Center, Leiden, The Netherlands; ^2^ Department of Clinical Oncology, Leiden University Medical Center, Leiden, The Netherlands; ^3^ Department of Medical Statistics and Bioinformatics, Leiden University Medical Center, Leiden, The Netherlands; ^4^ Institute of Mathematics, Leiden University, Leiden, The Netherlands

**Keywords:** CD8+ T cells, HLA class I, HLA-E, non-small cell lung cancer, survival

## Abstract

**INTRODUCTION:**

Tumor-infiltrating CD8+ T cells are associated with improved clinical outcomes in non-small cell lung cancer (NSCLC). Here we studied their prognostic effect in the context of the expression of HLA molecules that are key in tumor recognition (HLA-A, B and C) or suppression of immunity (HLA-E) as this is still unknown.

**METHODS:**

Tumor tissue of 197 patients with resected pulmonary adenocarcinoma was analyzed for the presence of CD8+ T cells and the expression of β2-microglobulin, HLA-A, HLA-B/C and HLA-E. The relation of these parameters with overall survival (OS) was assessed.

**RESULTS:**

Loss and low expression of HLA-A or HLA-B/C was found in 44% and 75% of cases respectively. A high CD8+ tumor infiltration was strongly associated with clinical benefit only when the tumors retained good expression of HLA-A and HLA-B/C (p=0.004). In addition, more than 70% of the tumors were found to display a high expression of HLA-E. The expression of HLA-E by tumor cells was an independent negative prognostic factor for OS (p=0.031). Importantly, a dense stromal CD8+ T cell infiltration was strongly associated with improved OS only in HLA-E negative tumors (p=0.005) and its prognostic effect was completely abolished when tumors highly expressed HLA-E (p=0.989).

**CONCLUSIONS:**

CD8+ T cell infiltration strongly contributes to a better prognosis in NSCLC when the tumor cells retain the expression of classical HLA class I and do not express HLA-E. Therefore, analysis of HLA-A, -B/C and HLA-E expression should be included as biomarkers to predict the response to immunotherapy.

## INTRODUCTION

Non-small cell lung cancer (NSCLC) is a leading cause of death globally [1–3]. The reported overall 5-year survival is 17% [2, 4], indicating the need for therapies that extend survival and provide a better quality of life. T-cell based immunotherapies hold great promise as a powerful new approach to treat NSCLC as treatment with antibodies interrupting immune checkpoint PD-1/PD-L1 has shown great clinical benefit in NSCLC [5–7]. The programmed death 1 (PD-1) receptor blocking antibody nivolumab was recently approved by the U.S. Food and Drug Administration to treat metastasized squamous NSCLC [8].

T-cell based immunotherapy of cancer is highly dependent on the presentation of tumor-specific antigens in the context of human leukocyte antigen (HLA) class I or class II molecules to tumor-infiltrating T cells (TILs) [9]. In NSCLC, the density of TILs, in particular the number of stromal CD8+ T cells, have strong prognostic value [10–15]. The expression of the classical HLA class I molecules A, B and C in NSCLC, however, frequently is down regulated [16, 17] and was found to affect overall survival (OS) [18]. Remarkably, studies on the interaction between CD8+ T cell infiltration and the expression of classical HLA class I are limited to one study showing that loss of HLA class I is associated with a sparser T-cell infiltrate [19].

The non-classical HLA class I molecules E, F and G can also be expressed by cancer cells. HLA-G expression was associated with limited lymphoid infiltration and poor prognosis in NSCLC [20], potentially via increased regulatory T-cell activity [21]. The expression of HLA-F, acting via the immune inhibitory receptors ILT-2 and ILT-4 [22], also had a negative impact on the prognosis of NSCLC patients [23]. HLA-E, which binds to the inhibitory CD94/NKG2A receptor expressed by activated NK cells and CD8 T cells, can directly suppress innate and adaptive immunity when expressed by cancer cells [24, 25]. We have studied the expression of HLA-E in different cancers [26, 27] and found that the beneficial prognostic effect of infiltrating CTLs in ovarian cancer was thwarted by high expression of HLA-E [27]. However, the expression and prognostic effect of HLA-E in NSCLC has not been studied.

To investigate the prognostic value of CD8+ tumor infiltrating T cells in the context of HLA-A, B and C as well as HLA-E and its association with OS, we retrospectively studied a group of 197 patients with NSCLC. We exclusively focused on pulmonary adenocarcinoma not only because this is the main histological subtype in NSCLC [1, 28] but also because HLA loss has been reported to be less frequent than in squamous cell carcinoma, the other major subtype of NSCLC [16–19, 29] and therefore is expected to benefit the most from active T-cell-mediated immunotherapy. Our study revealed that the expression of HLA-E by tumor cells was an independent prognostic factor for OS. High expression of HLA-E neutralized the positive prognostic value of high stromal CD8+ T cell infiltration in NSCLC.

## RESULTS

### Stromal CD8 T-cell infiltration correlates best with overall survival

A cohort of 197 patients with pulmonary adenocarcinoma was evaluated. The grade of differentiation by the tumor was classified as either poor (50%), moderate (33%) or well differentiated (17%). In 31% of cases, patients had advanced disease (stage III/IV) despite being classified as stage I/II based on pre-operative diagnostic modalities (Table 1). Mean age was 66 years (range 37- 90 years) and the number of males (n=99) and females (n=98) was evenly distributed.

**Table 1:** Overview of stage, differentiation and immunohistochemical expression patterns in pulmonary adenocarcinoma

Surgical-pathological staging (number, %)		
I	62	(31 %)
II	74	(38 %)
III	35	(18 %)
IV	26	(13 %)
Differentiation (number, %)		
Poor	98	(50 %)
Moderate	66	(33 %)
Well	33	(17 %)
β2-M (number, %)		
Low	47	(24 %)
High	150	(76 %)
HLA-A (number, %)		
Low	87	(44 %)
High	110	(56 %)
HLA-B/C (number, %)		
Low	148	(75 %)
High	49	(25 %)
HLA-E (number, %)		
Low	55	(28 %)
High	142	(72 %)
Total CD8+ (number, %)		
Low	96	(59 %)
High	68	(41 %)
CD8+ in tumor (number, %)		
Low	104	(64 %)
High	59	(36 %)
CD8+ in stroma (number, %)		
Low	92	(56 %)
High	71	(44 %)

**Figure 1:**
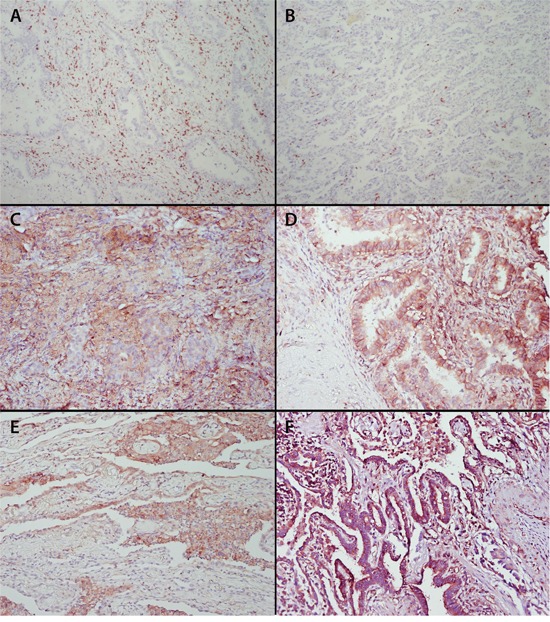
Staining of tumor infiltrating CD8+ T cells, β2-microglobulin, HLA-A, HLA-B/C and HLA-E in pulmonary adenocarcinoma Formalin-fixed, paraffin embedded tumor specimens of 197 non-small cell lung cancer patients were cut in 4 μm sections and immunohistochemically stained for CD8, β2-microglobulin, classical HLA-A and HLA-B/C, as well as non-classical HLA-E. According to the Ruiter scoring system [46] both the intensity and percentage of cells stained were assessed and expression was categorized as low (score 1-4) and high (score 5-9). Examples are shown of high **A.** and low **B.** stromal and intraepithelial CD8+ T cell infiltration; tumor with high β2-microglobulin expression **C.** examples of HLA-A **D.** HLA-B/C **E.** and HLA-E **F.** staining. Original magnification x200.

**Figure 2:**
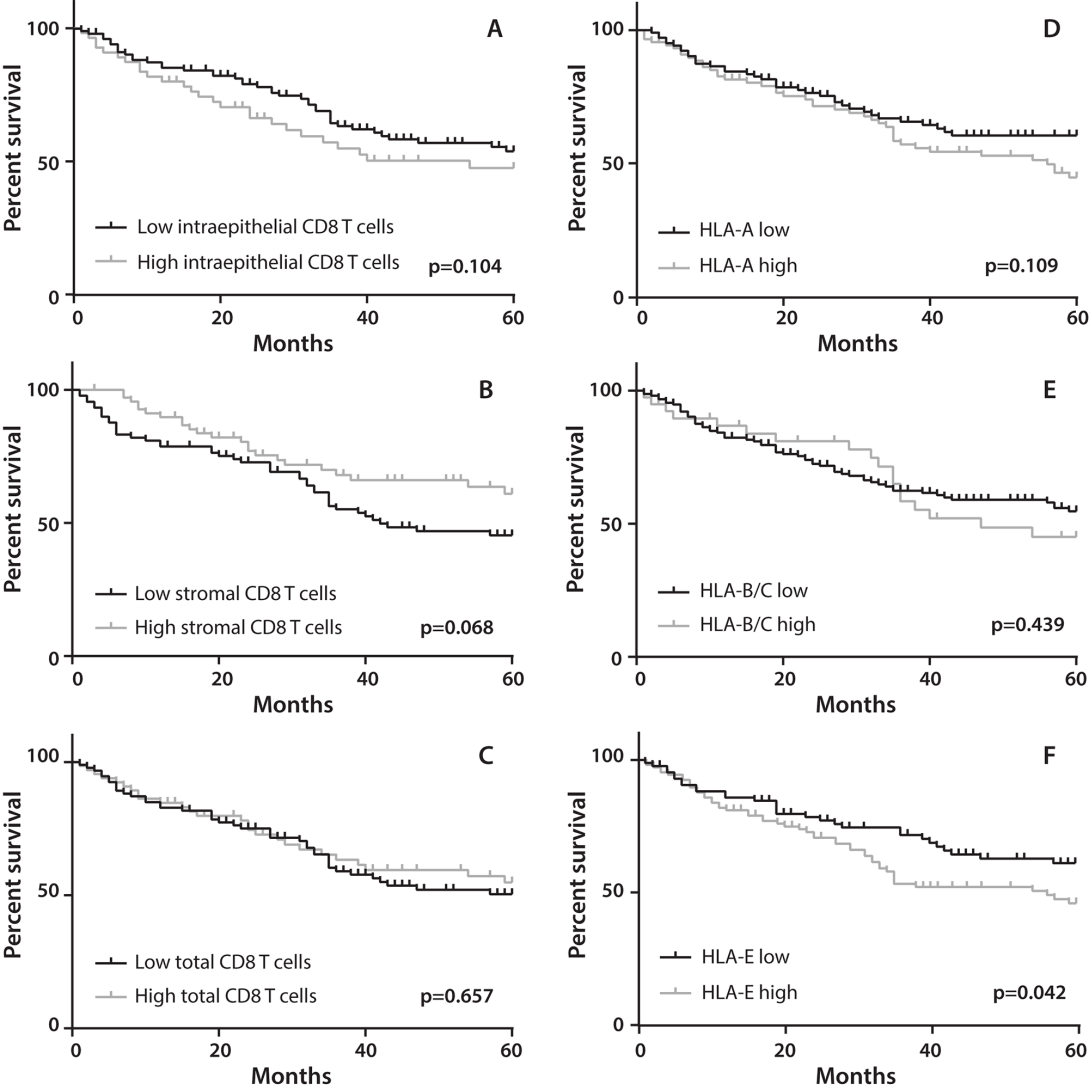
Association of CD8+ T cell infiltration and HLA expression with overall survival (OS) Patients were divided in two groups with low or high CD8+ T cell infiltration, based on the mean CD8+ T-cell count for all patients or on the basis of HLA expression. OS was defined as date of surgery until date of death due to any cause, or date of last follow-up with a maximum follow-up time of 5 years. Kaplan-Meier curves were used to estimate OS of the two groups whereas the log-rank test was used to compare the difference between the two curves. Survival curves are presented for **A.** patients with low (n=104) or high (n=59) intraepithelial CD8+ T cells (mean CD8+ cell count 194 cells/mm2 tumor) ; **B.** low (n=92) or high (n=71) stromal CD8+ T cells (mean CD8+ cell count 348 cells/mm2 tumor) ; **C.** low (n=95) or high (n=68) total CD8+ T cells (mean CD8+ cell count 271 cells/mm2 tumor). Furthermore, survival curves are presented for functional (i.e. positive staining for both HLA and β2-M) expression of **D.** HLA-A low (n=106) and high (n=91) ; **E.** HLA-B/C low (n=156) and high (n=41) ; **F.** HLA-E low (n=87) and high (n=110). A significant correlation (p=0.042) was observed between low HLA-E expression and improved survival (F).

The extent of CD8+ T-cell infiltration was studied by enumeration of intraepithelial and stromal CD8+ T cells in tumor sections. Examples of representative immunohistochemical stainings of CD8+ T cells are displayed in Figure 1. Overall intraepithelial CD8+ T-cell infiltration ranged from 7 to 1460 cells/mm^2^ tumor (mean 194; median 150), stromal CD8+ T cells from 35 to 1332 cells/mm^2^ tumor (mean 348; median 320) and total CD8+ T cells from 32 to 1008 cells/mm^2^ tumor (mean 271; median 246). There were no differences in total CD8+ T-cell tumor infiltration between males and females (chi square test, p=0.267). Patients were divided in two groups with low or high CD8+ T cell infiltration, based on the mean CD8+ T-cell count for all patients, and the association with OS was plotted. A relatively strong stromal CD8+ T-cell infiltration displayed the best association with a beneficial clinical outcome (log-rank test, p=0.068; Figure 2A–2C). The negative effect of low stromal CD8+ T-cell infiltration was magnified when the patients were divided on the basis of tertiles, with patients in the lower tertile defined as having low CD8+ stromal T cell infiltration and the other patients as having high stromal CD8+ T cell infiltration (p=0.046, Supplementary Figure S1), similar to what was reported before [10–14].

### Interaction between classical HLA class I expression and CD8+ T cells

Assessment of the expression of classical HLA class I molecules was performed using antibodies against β2-M, HLA-A and HLA-B/C (Figure 1). β2-M was expressed in 76% of cases, but HLA-A and HLA-B/C were expressed in only 56% and 25% of the cases, respectively (Table 1).

Subsequently, the association between tumor stage, HLA class I molecules and CD8+ T cell infiltration was assessed (Supplementary Table S1). High expression of HLA-A strongly correlated with high expression of HLA-B/C (p=0.0001). A clear correlation existed between the presence or absence of functional HLA class I expression and the total number of tumor-infiltrating CD8+ T cells. Tumors with downregulation of HLA-A (p=0.012) or HLA-B/C (p=0.018) displayed on average lower numbers of total tumor-infiltrating T cells (Supplementary Table S1 and Supplementary Figure S2).

**Figure 3:**
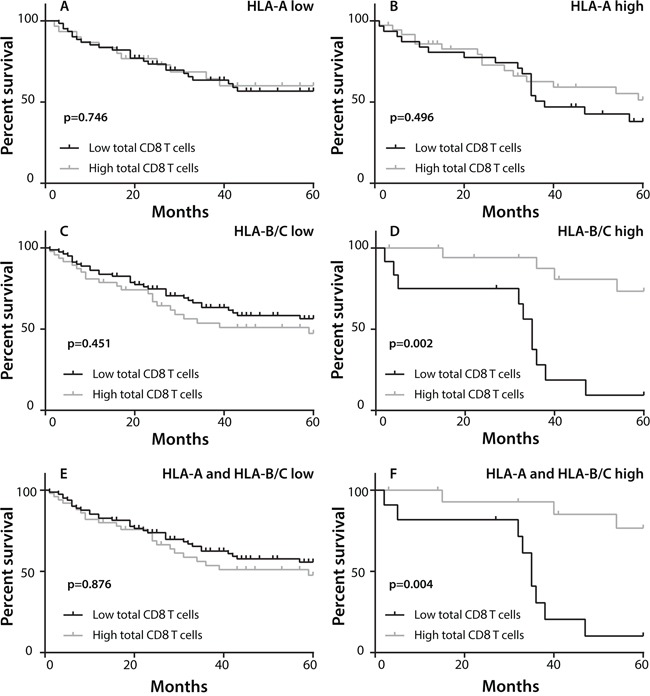
Effect of classical HLA class I expression and CD8+ T cell infiltration on overall survival (OS) Patients were divided in two groups with low or high CD8+ T cell infiltration, based on the mean CD8+ T-cell count for all patients and on the expression of classical HLA class I. Kaplan-Meier curves were constructed to estimate OS of the two groups whereas the log-rank test was used to compare the difference between the two curves. **A, B.** Comparison of OS between patients with low or high total CD8+ T cell infiltration in the context of low (A; n=63 vs 30, respectively) or high (B; n=32 vs 38, respectively) HLA-A expression. **C, D.** Comparison of OS between patients with low (n=83) or high (n=47) CD8+ T cell infiltration of whom the tumors displayed low HLA-B/C expression (C). Comparison of OS between patients with HLA-B/C positive tumors and high (n=21) or low (n=12) total CD8+ T cell infiltration (D). **E, F.** Comparison of OS between patients with low (n=62) or high (n=27) CD8+ T cell infiltration in tumors with low expression of both HLA-A and HLA-B/C (E). Comparison of OS between patients with low (n=11) or high (n=18) total CD8+ T cell infiltration in the context of tumors with high expression of both HLA-A and HLA-B/C. (F)

When patients were grouped according to a low or high expression of HLA-A or HLA-B/C, Kaplan Meier curves did not reveal any direct impact of classical HLA class I expression on clinical outcome (Figure 2D and 2E). However, an interaction analysis between classical HLA expression and total CD8+ T cell infiltration in tumor tissue revealed a clear beneficial effect of a dense CD8+ T cell infiltration in HLA-B/C positive tumors (HR 0.212, 95% CI 0.074-0.606, p=0.004) or HLA-A and HLA-B/C-positive tumors (HR 0.215, 95% CI 0.069-0.673, p=0.008) with respect to OS (Table 2 and Figure 3). This was not the case when CD8+ T-cell infiltration was analyzed in the context of HLA-A expression only. These results indicate that the presence of high numbers of CD8+ T cells is correlated with a favorable prognosis when classical HLA class I expression by the primary tumor is retained.

**Table 2:** Univariate and multivariate cox proportional hazard analysis

Variable		Univariate analysis		Multivariate analysis		
		HR	(95% CI)	p value	HR (95% CI)	p value
Stage	I/II vs III/IV	0.619	(0.399 - 0.961)	**0.033**	0.587 (0.377-0.913)	**0.018**
Sex	Male vs Female	1.834	(1.184 - 2.839)	**0.007**	1.785 (1.152-2.765)	**0.009**
Differentiation	poor vs medium/well	1.423	(0.928 - 2.182)	0.106		
β2-microglobulin	low vs high	0.762	(0.442 - 1.314)	0.328		
HLA-A	low vs high	0.703	(0.462 - 1.084)	0.112		
HLA-B/C	low vs high	0.822	(0.498 - 1.358)	0.443		
HLA-E	low vs high	0.632	(0.406 - 0.984)	**0.042**	0.612 (0.392-0.956)	**0.031**
Intraepithelial CD8	low vs high	0.682	(0.427 - 1.087)	0.108		
Stromal CD8	low vs high	1.560	(0.962 - 2.530)	0.072	1.613 (0.993-2.620)	0.054
Total CD8	low vs high	1.130	(0.705 - 1.812)	0.659		
HLA-E low	high vs low stromal CD8	0.303	(0.124 - 0.741)	**0.009**		
HLA-E high	high vs low stromal CD8	1.004	(0.550 - 1.835)	0.989		
Stromal CD8 high	high vs low HLA-E	3.282	(1.308 - 8.232)	**0.011**		
Stromal CD8 low	high vs low HLA-E	1.032	(0.585 - 1.818)	0.914		
HLA-B/C high	high vs low total CD8	0.212	(0.074 - 0.606)	**0.004**		
HLA-A and B/C high	high vs low total CD8	0.215	(0.069 - 0.673)	**0.008**		

Significant differences (p<0.05) are indicated in bold.

### HLA-E expression is a strong negative determinant for OS

**Figure 4:**
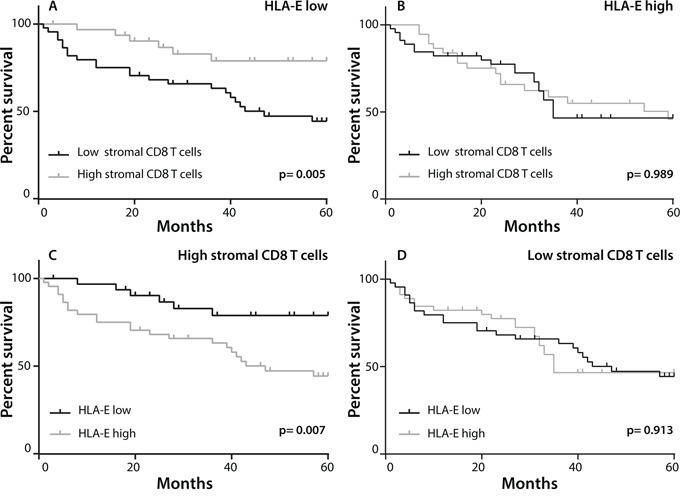
Prognostic benefit in HLA-E negative tumors with high CD8+ T cell infiltration Patients were divided in two groups with low or high stromal CD8+ T cell infiltration, based on the mean CD8+ T-cell count for all patients and on the expression of HLA-E. Kaplan-Meier curves were constructed to estimate OS of the two groups whereas the log-rank test was used to compare the difference between the two curves. **A.** The effect of CD8+ T cell infiltration in patients with low HLA-E expression (n=77), showing that a high stromal CD8+ T cell infiltration was strongly associated with a better OS. **B.** The effect of a high stromal CD8+ T cell infiltrate was neutralized in tumors with high HLA-E expression (n=86). **C, D.** Conversely, in patients with high stromal CD8+ T cell influx (n=71), a high HLA-E expression is associated with a worse OS (C). In patients with low presence of stromal CD8+ T cells (n=92), HLA-E expression had no effect on OS (D).

In more than 70% of pulmonary adenocarcinoma cases a high expression of HLA-E was observed (Figure 1F and Table 1). In 20.3% of these tumors displaying a functional HLA-E molecule, no expression of HLA-A and HLA-B/C was observed. The high expression of HLA-E was associated with worse OS (HR 0.632, 95% CI 0.406-0.984, p= 0.042; Table 2 and Figure 2F). Since both stromal CD8+ T-cell infiltration and the expression of HLA-E displayed the strongest effects on overall survival as a single determinant (Figure 2B and 2F, Supplementary Figure S1), a subsequent analysis was performed to study the interaction between these two factors. Clearly, a dense stromal CD8+ T cell infiltration showed a strong positive prognostic value in HLA-E negative tumors (HR 0.303, 95% CI 0.124-0.741, p=0.009; Figure 4A and 4B). However, this beneficial effect of a dense stromal CD8+ T cell infiltration disappears in patients with high expression of HLA-E (HR 1.004, 95% CI 0.550-1.835, p=0.989; Figure 4C and 4D). In conclusion, the beneficial effect displayed by tumor-infiltrating stromal CD8+ T cells is impeded when HLA-E is highly expressed by tumors.

### HLA-E expression is an independent determinant of OS in pulmonary adenocarcinoma

In order to assess the effect of each single variable on the relative risk of death, univariate and multivariate Cox proportional hazards analysis were performed to quantify survival differences (Table 2). Tumor stage and male gender have been reported before as negative risk factors for OS in pulmonary adenocarcinoma [30] and indeed in our cohort high stage tumors (stage I/II vs stage III/IV, HR 0.619, 95% CI 0.399-0.961, p=0.033) as well as male gender (HR 1.834, 95% CI 1.184-2.839, p=0.007) were associated with worse OS. In the univariate analysis, a low expression of non-classical HLA-E by tumor cells was associated with a strong reduced risk of death in this cohort (HR 0.632, 95% CI 0.406-0.984, p=0.042). Presence of high stromal CD8+ T cells correlated with improved OS and reached near-significance (HR 1.560, 95% CI 0.962-2.530, p=0.072) and hence was included in the multivariate analysis together with tumor stage, gender and HLA-E expression.

Similar to the univariate analysis the positive effect of stromal CD8+ T cells on OS approached statistical significance (HR 1.613, 95% CI 0.993-2.620, p=0.054) in the multivariate analysis. In addition to tumor stage and gender, the increased expression of HLA-E was significantly associated with OS (HR 0.612, 95% CI 0.392-0.956, p= 0.031) indicating that low HLA-E expression is an independent positive prognostic factor for OS in pulmonary adenocarcinoma.

## DISCUSSION

The infiltration of NSCLC by CD8+ T cells is positively associated with a longer OS irrespective of tumor stage [10–15] and our study confirms this association of CD8+ T-cell infiltration with better OS. It is important to identify the most important factors governing a successful attack of NSCLC by CD8+ T cells as illustrated by the facts that a) more than 40% of NSCLC patients respond to checkpoint inhibitor therapy [5–7]; and b) especially those patients are likely to respond in whom the tumor has generated neo antigens for CD8+ T cells [31]. One of the key molecules in this process is the expression of HLA molecules required to present tumor-specific peptides to T cells. When measured with a pan-HLA class I antibody, the loss of HLA is observed in almost half of the patients with pulmonary adenocarcinoma [16–19, 32]. We used antibodies to distinct the expression of HLA-A and HLA-B/C in order to chart the HLA loss in more detail (Table 1). We found that HLA-A was decreased in about 40% of the patients while the decrease in HLA-B/C expression was even as high as 75% which is in line with only one other study that reports specifically on loss of HLA-B/C in NSCLC [33]. In addition, interaction analyses of HLA expression and CD8+ T-cell infiltration led to the novel observation that the prognostic effect of a dense CD8+ T-cell tumor infiltration is only retained when tumors display a high expression of classical HLA class I, in particular HLA-B/C (Figure 3).

Other key molecules governing a successful attack of T cells in NSCLC are the so-called checkpoints [34]. The non-classical HLA-E molecule is the ligand for the inhibition receptor CD94/NKG2A and represents an important immunologic checkpoint [24, 25]. This study is the first to show that a high expression of the non-classical HLA-E molecule affects overall survival in NSCLC. Both univariate and multivariate analysis revealed high HLA-E expression by tumor cells as an independent predictor of poor prognosis (Table 2 and Figure 2). The expression of HLA-E can inhibit the function of T lymphocytes and natural killer (NK) cells when it engages with CD94/NKG2A [24, 25, 35], as well as activate these cells when HLA-E engages with CD94/NKG2C [36]. A few studies in breast cancer and cervical adenocarcinoma have reported survival benefit for HLA-E expressing tumors [37, 38] while others, similar to us, reported a negative effect of HLA-E on OS in ovarian cancer, colorectal cancer and gastric cancer [27, 39–41]. Potentially, the type of receptor for HLA-E expressed by CD8 T cells is at the basis of this difference. In ovarian cancer and colorectal cancer the T cells were shown to express the inhibitory receptor CD94/NKG2A [27, 39]. In line with previous studies in NSCLC, a dense stromal CD8+ T-cell tumor-infiltrate was associated with longer OS (Figure 2 and Supplementary Figure 1) [10–15]. In our study, a high expression of HLA-E by tumor cells clearly had a negative effect on CD8+ T cells. The positive prognostic effect of stromal CD8+ T cells on OS was only apparent in patients with low expression of HLA-E on their tumor cells. A high tumor expression of HLA-E completely abolished the prognostic effect of CD8+ T-cell infiltrate (Table 2 and Figure 4).

Our study on the interaction between tumor expressed HLA and CD8+ T-cell infiltration in relation to survival raises two important issues. Currently, CD8+ T-cell infiltration and expression of PD-L1 are being considered as biomarkers to select the population of NSCLC patients that are most likely to respond to checkpoint antibody therapy [42]. Based on our data showing that the prognostic effect of CD8+ T cells is most pronounced in those tumors still expressing HLA-B/C, one could consider including the analysis of classical HLA class I expression in this selection procedure. The second issue concerns the improvement of current PD-1/PD-L1 therapy or alternative checkpoint treatment strategies in NSCLC. Our results showed that about 70% of the pulmonary adenocarcinomas displayed a high expression of HLA-E (Table 1). In view of its effect on both T cells and NK cells, blocking HLA-E and/or its CD94-NKG2A inhibitory receptor may form a valuable target for the immunotherapy of NSCLC. Treatment with anti-NKG2A monoclonocal antibody was shown to overcome HLA-E mediated suppression of anti-tumor cellular cytotoxicity *in vitro* [43, 44] and this has resulted in a currently ongoing phase I/II trial in which patients with advanced head and neck cancer are treated with an anti-NKG2A monoclonocal antibody (ClinicalTrials.gov, Identifier: NCT02331875). Potentially, a combination of antibodies to these two different checkpoints may even have a synergistic effect.

In conclusion, our results confirm the pivotal protective role of tumor infiltrating CD8+ T cells in NSCLC and in addition show that their effect is particularly apparent when the tumor cells retain the expression of classical HLA class I and do not express the non-classical molecule HLA-E. These results warrant the inclusion of HLA-A, -B/C and HLA-E as biomarkers to predict the response to immunotherapy and the use of HLA-E or NKG2A blocking antibodies for the treatment of NSCLC.

## MATERIALS AND METHODS

### Study population

We retrospectively identified 197 patients diagnosed with non-small cell lung cancer (NSCLC), subtype adenocarcinoma, in the Leiden University Medical Center (LUMC) between 2000 and 2013. All patients underwent preoperative staging and were classified as stage I/II NSCLC and subsequently underwent surgical resection of the primary tumor with systematic lymph node dissection. After surgical removal of the tumor and its draining lymph nodes, patients were considered disease free. Tumor tissue, clinical data and follow-up data were collected from all patients. Staging of NSCLC was determined according to the TNM (Tumor, Node, Metastasis) classification using the updated guidelines of the International Association for the Study of Lung Cancer (IASLC) [45]. The use of archival tumor blocks was in accordance with guidelines from the Dutch Federation of Medical Research Association. Since this retrospective study does not fall under the scope of the Medical Research Involving Human Subjects Act (WMO), it was not subject to a prior review by a Medical Ethical Committee and written informed consent was not obtained. However, patient data were anonymized.

### Antibodies

Mouse monoclonal antibodies HCA-2 (anti HLA-A, 1:1000) and HC-10 (anti HLA B/C, 1:500) were used to detect expression of the free heavy chain of the HLA class I molecule. Rabbit anti-human β2-microglobulin (anti-β2M; clone A-072, DAKO, 1:2000) and mouse anti-human HLA-E (clone MEM-E/02; Serotec, Germany [1:200]) antibodies were used in order to detect the light chain and non-classical HLA-E heavy chain respectively. Mouse monoclonal CD8 antibody (clone IA5, Leica Biosystems, Germany [1:500]) was used for the detection of the CD8+ T-cells.

### Immunochemistry

Formalin-fixed, paraffin embedded tumor blocks were cut in 4 μm sections using a microtome and deparaffinized in xylene. The endogenous peroxidase activity was blocked for 20 minutes using 0.3% hydrogen peroxide/methanol. The samples were subsequently rehydrated in 70% and 50% ethanol and antigen retrieval was performed by heating the samples to 97°C for 10 minutes in citrate buffer (either pH 9.0 or pH 6.0, DAKO, Glostrup, Denmark). Antibodies were diluted in phosphate buffered saline (PBS, Fresenius Kabi Bad Homburg, Germany) with 1% bovine serum albumin (BSA) and incubated overnight at room temperature. The slides were stained immunohistochemically with horseradish peroxidase (HRP)-conjugated anti-mouse IgG (DAKO envision) for 30 minutes at room temperature. NovaRed (Vector, Burlingame, USA) was applied as a chromagen followed by counterstaining with Mayer's hematoxylin (Klinipath). All washing steps were done with PBS. All slides were mounted with Pertex mounting medium (HistoLab, Sweden).

The microscopic evaluation and analysis of the HCA2, HC10, β2M and HLA-E staining was performed by two independent observers without prior knowledge of clinical or histopathological parameters (observer one 100% of the cohort, observer two 20% of the cohort). The inter-observer agreement was assessed by calculating Cohen's kappa coefficient resulting in a coefficient of >0.70 for all stainings which indicates a substantial inter-observer agreement.

The grade of tumor differentiation was determined and classified as either poorly differentiated, moderately differentiated or well differentiated based on the immunohistochemically stained slides. Expression patterns of the previously mentioned antibodies were assessed according to the scoring system proposed by the Ruiter *et al* [46]. Using this method the entire slide is screened and the percentage of positive tumor cells was classified as: absent 0%, sporadic 1-5%, local 6-25%, occasional 25-50%, majority 51-75% and large majority 76-100% (1-6). Furthermore, this score includes intensity of the staining which is classified as negative, low, medium and high (0-3). The intensity was noted for all antibodies with the exception of CD8 since high intensity was always observed. The final score was based on both intensity and percentage and was categorized as 1-4 (low expression) and 5-9 (high expression).

### Quantification of infiltrating CD8+ T-cells

CD8+ T-cell infiltration was assessed by screening five randomly captured high resolution (200X) images of each slide. The area of the tumor nests and stromal areas were marked and calculated using NIH-ImageJ software (v1.48). CD8+ T cells were counted by area and represented as the number of cells per mm2 of tumor area with a distinction between intraepithelial and stromal CD8+ T cells. The mean number of intraepithelial, stromal and total number of tumor-infiltrating CD8+ T cells were calculated and patients were dichotomized for high or low CD8+ T cell infiltration based on the mean CD8+ T cell infiltration for all patients.

### Statistical analysis

Nonparametric Mann–Whitney *U* test was used to compare continuous variables between patient groups and group comparisons of categorical data were performed by two-tailed χ2 test. Overall survival (OS) was defined as date of surgery until date of death due to any cause, or date of last follow-up with a maximum follow-up time of 5 years. When assessing survival based on HLA expression, low and high expression of HLA indicates the presence of a functional HLA molecule, i.e high expression of both β2M and the HLA heavy chain of HLA-A, HLA-B/C and HLA-E respectively. Survival was estimated by using Kaplan—Meier methodology and the log-rank test was used to compare the two curves. Univariate Cox proportional hazards model was used to study the effect of single determinants on OS. Multivariate Cox regression analysis was performed with variables that reached statistical significance in univariate analysis. Stepwise regression was employed to estimate the final model. Two-sided P values of <0.05 were considered statistically significant. Bonferroni correction was applied for multiple testing. Statistical software package SPSS 20.0 (SPSS, Chicago, IL) was used for data analysis. GraphPad Prism 6.02 (Graphpad Software, LA Jolla, CA) was used to estimate survival curves.

## SUPPLEMENTARY FIGURES AND TABLES



## Abbreviations

β2M: β2-microglobulin
BSA: Bovine Serum Albumin
HLA: Human Leucocyte Antigen
HRP: Horseradish Peroxidase
LICR: Ludwig Institute for Cancer Research
LUMC: Leiden University Medical Center
NK: Natural Killer
NSCLC: Non-Small Cell Lung Cancer
OS: Overall survival
PBS: Phosphate Buffered Saline
PD-1: programmed death 1
TIL: Tumor-infiltrating lymphocyte
