# Supplementary material for: The positive prognostic effect of stromal CD8+ tumor-infiltrating T cells is restrained by the expression of HLA-E in non-small cell lung carcinoma
Source: Oncotarget. 2015 Dec 2;7(3):3477–88. doi: 10.18632/oncotarget.6506 (PMC4823121; doi:10.18632/oncotarget.6506)
Supplement: Supplementary file 1 [file oncotarget-07-3477-s001.pdf]

SUPPLEMENTARY FIGURES AND TABLE

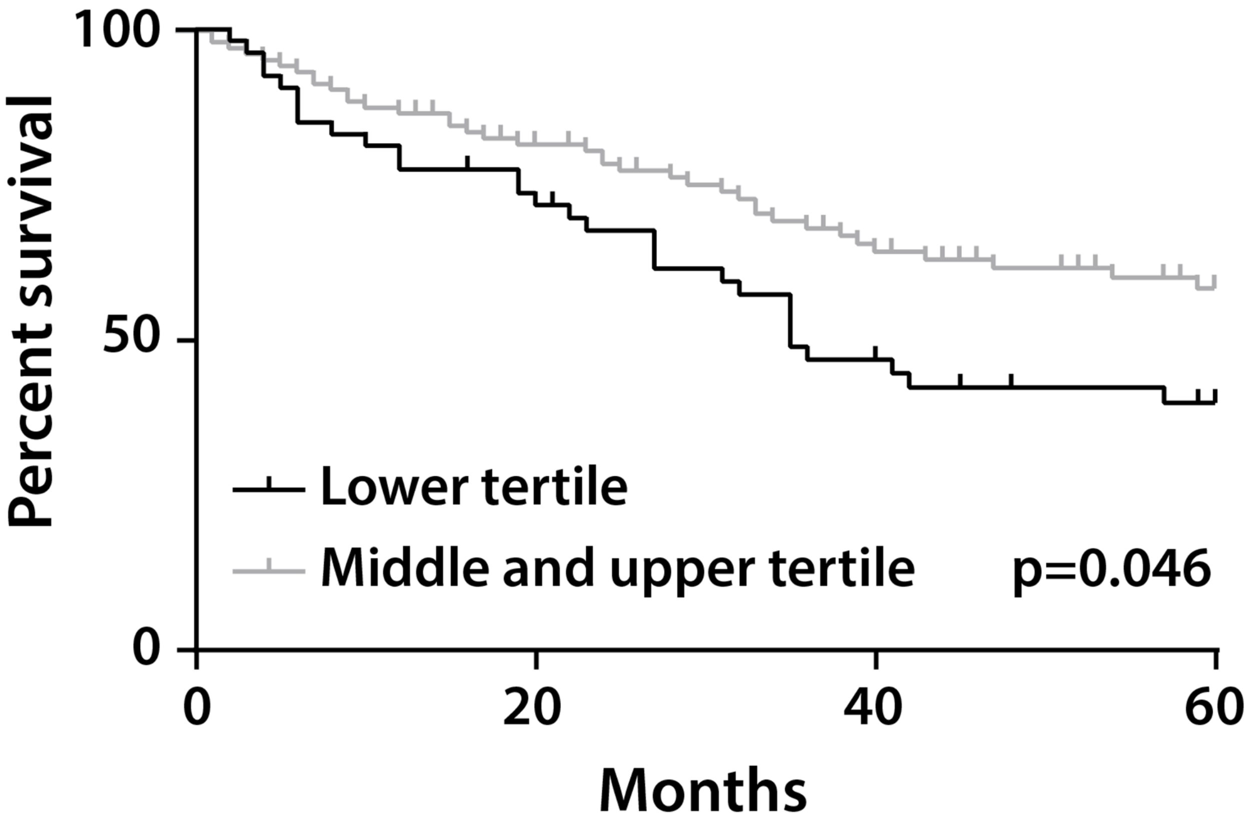

Supplementary Figure S1: Tertile based grouping of stromal CD8+ T cells and influence on OS. The patients were grouped based on the CD8+ T cell counts/mm^2 tumor. Patients with CD8 T cell counts belonging to the lower tertile were categorized as low (n = 54) whereas patients with counts in the middle and upper tertile were categorized as high (n = 109). Kaplan-Meier curves were used to estimate OS whereas the log-rank test was used to compare the differences between the two curves (p = 0.046).

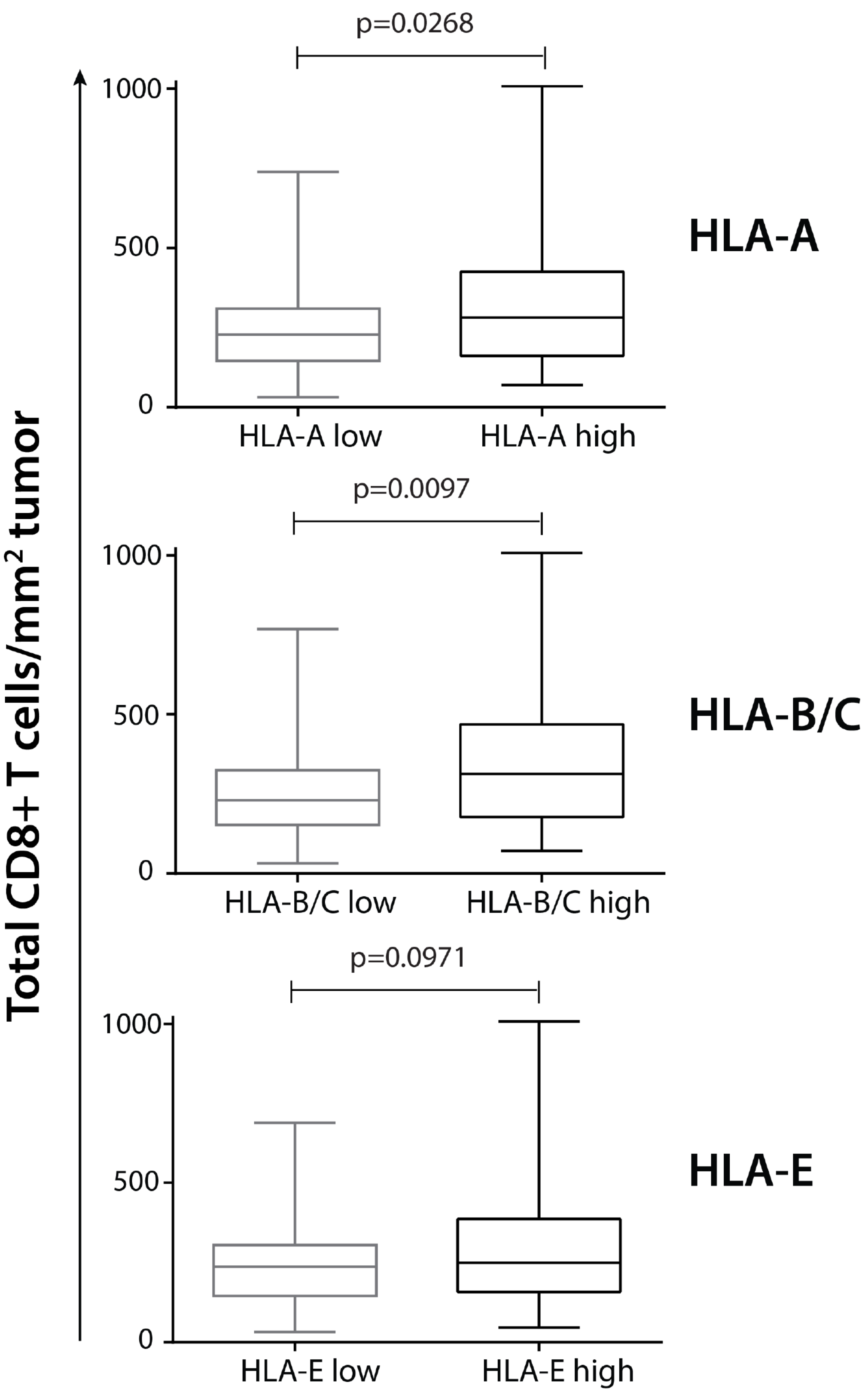

Supplementary Figure S2: HLA expression and its relation with total CD8+ T cell infiltration in the primary tumor. The association between the total CD8+ T cell count per mm^2 tumor and expression of HLA-A (low vs high, n = 106 vs n = 91), HLA-B/C (low vs high, n = 156 vs n = 41) and HLA-E (low vs high, n = 87 vs n = 110) was assessed. Tumors expressing classical HLA are infiltrated with higher numbers of CD8+ T cells (Mann–Whitney U test, $p < 0.05$).

Supplementary Table S1: Relationship of tumor characteristics with HLA expression and CD8+ T cell expression in pulmonary adenocarcinoma

	HLA-A		P value	HLA-B/C		P value	HLA-E		P value
	**High**	**Low**		**High**	**Low**		**High**	**Low**	
Stage									
I	36	26	0.621	17	45	0.872	44	18	0.777
II	43	31		16	58		56	18	
III	16	19		9	26		25	10	
IV	15	11		7	19		17	9	
β2-M									
Low	18	29	**0.007**	8	39	0.179	32	15	0.576
High	92	58		41	109		110	40	
HLA-A									
Low				6	81	**0.0001**	60	27	0.426
High				43	67		82	28	
HLA-B/C									
Low							106	42	0.856
High							36	13	
Total CD8+									
Low	41	55	**0.012***	16*	80	**0.018***	64	32	0.480*
High	45	23		25	43		53	15	
CD8+ in stroma									
Low	45	47	0.819*	19	73	0.444*	66	26	0.990*
High	41	30		22	49		51	20	
CD8+ in tumor									
Low	50	54	0.426*	21	83	0.186*	68	36	0.057*
High	36	23		20	39		49	10	

Significant results (p <0.050) are indicated in bold.

*Bonferroni corrected p value
